# Cytoplasmic 5′-3′ exonuclease Xrn1p is also a genome-wide transcription factor in yeast

**DOI:** 10.3389/fgene.2014.00001

**Published:** 2014-02-06

**Authors:** Daniel A. Medina, Antonio Jordán-Pla, Gonzalo Millán-Zambrano, Sebastián Chávez, Mordechai Choder, José E. Pérez-Ortín

**Affiliations:** ^1^Departamento de Bioquímica y Biología Molecular and ERI Biotecmed, Universitat de ValènciaBurjassot, Spain; ^2^Departamento de Genética and Instituto de Biomedicina de Sevilla, Hospital Universitario Virgen del Rocío/CSIC/Universidad de SevillaSeville, Spain; ^3^Faculty of Medicine, Department of Molecular Microbiology, Technion-Israel Institute of TechnologyHaifa, Israel

**Keywords:** transcription rate, nascent transcription, mRNA synthesis, mRNA decay, mRNA stability

## Abstract

The 5′ to 3′ exoribonuclease Xrn1 is a large protein involved in cytoplasmatic mRNA degradation as a critical component of the major decaysome. Its deletion in the yeast *Saccharomyces cerevisiae* is not lethal, but it has multiple physiological effects. In a previous study, our group showed that deletion of all tested components of the yeast major decaysome, including *XRN1*, results in a decrease in the synthetic rate and an increase in half-life of most mRNAs in a compensatory manner. Furthermore, the same study showed that the all tested decaysome components are also nuclear proteins that bind to the 5′ region of a number of genes. In the present work, we show that disruption of Xrn1 activity preferentially affects both the synthesis and decay of a distinct subpopulation of mRNAs. The most affected mRNAs are the transcripts of the highly transcribed genes, mainly those encoding ribosome biogenesis and translation factors. Previously, we proposed that synthegradases play a key role in regulating both mRNA synthesis and degradation. Evidently, Xrn1 functions as a synthegradase, whose selectivity might help coordinating the expression of the protein synthetic machinery. We propose to name the most affected genes “Xrn1 synthegradon.”

## Introduction

Xrn1 (also called Kem1 in yeast, Pacman in *Drosophila*, and XRN4 in *Arabidopsis*) is a pleiotropic eukaryotic protein (Kim and Kim, 2002) involved in several RNA processing and degradation processes, such as general mRNA decay pathways (Muhlrad et al., 1994, 1995), surveillance mechanisms for aberrant mRNAs (He et al., 2003) and tRNAs (Wichtowska et al., 2013), processing intron lariats after splicing and ncRNA processing and degradation (reviewed in Nagarajan et al., 2013). It is dispensable for viability in optimally proliferating yeast, *Drosophila* and *Arabidopsis*, but its absence brings about pleiotropic effects that relate specially with both development in higher eukaryotes and growth control in lower eukaryotes (Kim and Kim, 2002; Jones et al., 2012; Nagarajan et al., 2013). The specificity of *Arabidopsis* XRN4 (Rymarquis et al., 2011) and animal XRN1 (Orban and Izaurralde, 2005) on certain types of transcripts has led to the suggestion that its major role is not bulk decay, but the control of developmental programs. These general phenotypes have been related with the large number of mRNAs whose stability and levels are affected in *xrn1* mutants or with “downstream” indirect effects (Nagarajan et al., 2013).

The most studied role of Xrn1 is related with the degradation of decapped mRNAs as a final step in the mRNA life cycle. The 5′ mRNA decapping pathway is considered the main one for yeast eukaryotic mRNA turnover, and probably in other eukaryotes as well (reviewed in Parker, 2012). The presence of Xrn1 in the nucleus has been related with the processing of ncRNAs, such as snoRNA or tRNAs (revised in Nagarajan et al., 2013). However, we recently showed that Xrn1 shuttles between the cytoplasm and the nucleus, and plays a role as a transcriptional activator of a large number of yeast genes (Haimovich et al., 2013). Although it has been seen to affect specific sets of genes in other organisms, such as *Arabidopsis* (Rymarquis et al., 2011), *S. cerevisiae* (He et al., 2003) or *Drosophila* (Jones et al., 2012), a comprehensive analysis of its effects on mRNA synthesis and degradation rates is lacking.

Here we show that Xrn1 absence produces biased effects toward genes that are highly transcribed encoding unstable mRNAs. These genes are strongly enriched in the functional categories related to protein biosynthesis. These results support a role for Xrn1 in the mRNA homeostasis of genes whose functions are most important for optimized cell growth. We also show that Xrn1 functions in transcription at both the initiation and elongation levels, and that it probably is a component of a pathway that involves mRNA export proteins Yra1 and the nuclear pore complex Mlp1.

## Materials and methods

### Yeast strains and growth conditions

The yeast strains used in this work derive from a previous work (Solinger et al., 1999; Haimovich et al., 2013). We used a wild-type strain (*MAT*a, *ade2*, *ura3*-52, *XRN1*), a complete deletion mutant (*MAT*a, *ade2*, *ura3*-52, Δ*xrn1::URA3*), and a point mutant (*MAT*a, *ade2*, *ura3*-52, *xrn1*^*D*208*A*^). Cells were grown on YPD (2% glucose, 2% peptone, 1% yeast extract) overnight at 28°C. Samples were taken at OD_600_ 0.5.

### Chromatin immuno-precipitation (ChIP) and run-on experiments

ChIP and run-on experiments of the individual genes were carried out as previously described (Haimovich et al., 2013).

### Genome-wide assays

To determine mRNA half-lives (HL), we used the transcription shut-off assay, as previously described (Pelechano and Pérez-Ortín, 2008). Transcription was stopped by adding thiolutin at 5 μg/mL. Culture samples were taken at different time points. RNA samples were purified by hot phenol-chloroform extraction and were reverse-transcribed to cDNA in the presence of radioactive ^33^P-d[CTP]. Labeled cDNA was hybridized in in-house prepared nylon membranes macroarrays (Alberola et al., 2004) and the mRNA half-life was estimated by the decay of signal over time. The decay of the genome-wide HL was adjusted by the decay of a group of genes acquired by Northern blot. Only those genes which correlated well in the decay curve (R-Pearson > 0.6) and an HL below 200 min were considered confident values. Nascent transcription rate (nTR) and indirect mRNA stability were determined by Genomic Run-On (GRO) as previously described (García-Martínez et al., 2004). The total RNA for all the strains was determined by repeated acid-phenol extraction, as described elsewhere (García-Martínez et al., 2004). The proportion of [mRNA/Total RNA] was estimated by an Experion assay (Biorad) to normalize the mRNA levels (Haimovich et al., 2013). All the experiments were done in triplicate and the data were normalized using the ArrayStat statistics software (Imaging Research Inc.). The Xrn1 Chip-Exo and other genome-wide data were published in Haimovich et al. (2013), (GEO accession numbers GSE44312 and GSE29519).

### Responsiveness determination

The response effect on both the nTR and the HL was called *responsiveness*. Responsiveness was estimated from the ratio between the changes in the HL of the mutant in relation to the wild-type strain, and the change in nTR between the wild-type strain and the mutant. In this way, both ratios were higher than 1. The ratios (for the nTR and HL changes) were ranked for each gene regarding its position in the quintiles data (from 1 for the genes with the lowest ratio to 5 for the genes with a high ratio). Then, the rank of both ratios was summed. The data were classified as high responsiveness when their rank sum went over 8 points and as low responsiveness when the rank sum was lower than 3 points. Those genes with more than 8 points were strongly affected in both nTR and HL, whereas a gene below 3 points was almost unaffected.

### Gene ontology search and bioinformatics tools

The gene ontology (GO) searches for Biological Process and Cellular Component the different categories enriched in the changes of HL, nTR, Xrn1 binding and responsiveness were done using the online GOrilla tool (Eden et al., 2009), and the data obtained were filtered with the online ReViGO tool (Supek et al., 2011). To eliminate excessively broad GOs, the categories with over 12% of the whole gene set in the ReViGO filter were eliminated. The statistical analysis and some of the graphical representations were carried out using the R statistical language (R Development Core Team, 2008) and its packet LSD (Schwalb et al., 2011). Venn diagrams were drawn using the online BioVenn tool (Hulsen et al., 2008).

## Results

### Xrn1 inactivation extends most mRNA half-lives

Xrn1 is a 5-3′ exonuclease that is required for the main cytoplasmic mRNA degradation pathway (Muhlrad et al., 1994). It is not, however, an essential protein for yeast survival (reviewed in Nagarajan et al., 2013). It is assumed that it degrades many or most mRNAs, be it with some biases (He et al., 2003).

In this work, we used two different mutants of *XRN1*, a complete deletion (Δ*xrn1*) and a point mutation (D208A), which abolish its catalytic activity (Haimovich et al., 2013). In order to determine the potential preferences of Xrn1 activity, we performed two different genome-wide assays: a transcription shut-off with thiolutin, a drug that blocks RNA polymerase activity, followed by a macroarray hybridization of the successive chasing time points. This kind of assay is easy to perform, but entails a plethora of potential biases and drawbacks (Pérez-Ortín et al., 2012). This assay produces a direct measurement of mRNA half-lives, although the technical limitations of array hybridization and quantification, plus the need for a line adjustment of the decay curve, limits the number of confident values to less than 2000 genes (see Materials and methods). The second method is based on an indirect calculation of the degradation rate for each mRNA by assuming that it is equal to the synthesis rate (Pérez-Ortín et al., 2012, 2013) because a steady state for mRNA concentrations exists during yeast exponential growth in YPD (Pelechano and Pérez-Ortín, 2010). This approach provides a much larger HL set and has totally different biases (Pérez-Ortín et al., 2012). Another problem of this indirect calculation is the mathematical linkage between the calculated HLs and the parameters used for their calculation: nTRs and HLs (Pérez-Ortín et al., 2012). Accordingly, we used only thiolutin-based HLs in all our comparisons with nTR and mRNA levels. However by using both the HL datasets for the two independent mutants, it is possible to obtain reliable HLs values and to categorize genes accordingly.

**Figure 1:**
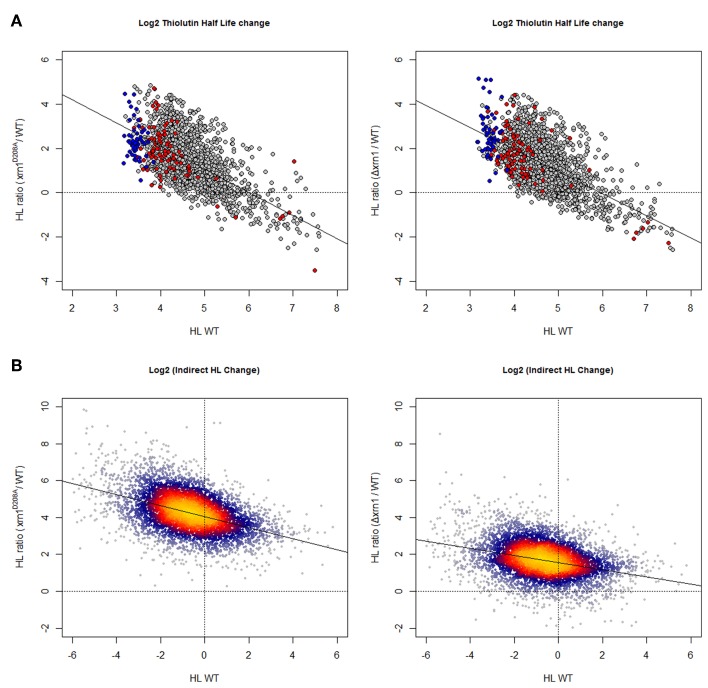
**Changes in mRNA HL due to disruption of *Xrn1* inversely correlate with mRNA stability. (A)** Plots of the ratios between mutant and wild-type HL against HL in the wild type (wt). The cloud shows 1915 data for the genes with confident HL measures in a shut-off with thiolutin in both wt and mutants (see Materials and methods for details). **(B)** The same kind of plots, but using 5400 data points from an indirect HL determination using the GRO protocol and mRNA level quantification by a macroarray analysis (García-Martínez et al., 2004). This method provides more data points, but they are mathematically linked to nTR and (mRNA). See the main text for discussion. The axes are on a log_2_ scale. Plots in **(A)** show genes for RP (blue) and RiBi (red) highlighted. Plots in **(B)** were done using the LSD packet (Schwalb et al., 2011) and show a color code proportional to gene density: from red (high) to blue (low).

As shown in Figure 1, and as expected, using both methods for HL determinations the deletion of Xrn1 or the disruption of its enzymatic activity led to the stabilization of most mRNAs. The disruption of Xrn1 had a stronger effect on unstable mRNA and resulted in a clear negative tendency in the plot of the HL changes vs. HL in wt. It seemed to have a stronger effect on the point mutation than on the deletion mutant.

**Figure 2:**
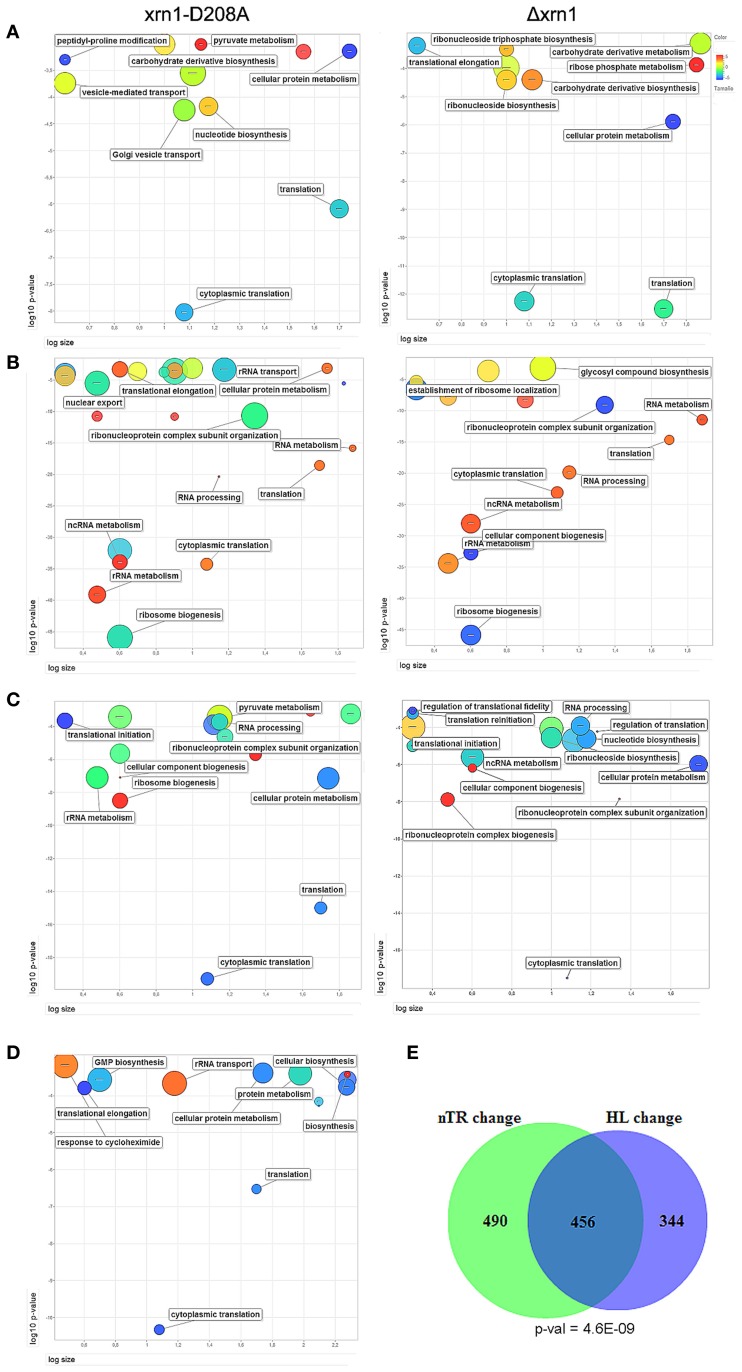
**ReViGO plots of the GO searches.** Plots of the statistically overrepresented GOs among genes in the highest 20% change in the mutant against wt in the thiolutin HL determination **(A)** or nTR **(B)**. A similar search for the genes with responsiveness ≥8 **(C)** and the genes bound by Xrn1 **(D)**. ReViGO plots use previous GOrilla results for the Biological Process and plot the GO categories according to their *p*-value (Y-axis), the number of genes in category (X-axis) and semantic spaces (color and size of circle). The complete list of the GO categories found for the GO Biological Process and Cellular Component is presented in Table S2. The large overlap and its *p*-value between the genes most affected (quintiles >3, see Materials and methods) for both mutants in nTR and HL the Δ*xrn1* is shown **(E)**.

When analyzing the list of genes that were most affected by Xrn1 inactivation, in all four cases (deletion and point mutant, direct and indirect HL measurements) we found a statistical enrichment of the GO categories of the growth-related genes, including protein biosynthesis. Other GOs were also overrepresented, specifically those related with carbohydrate derivative biosynthesis (see Table S1; Figure 2A).

**Figure 3:**
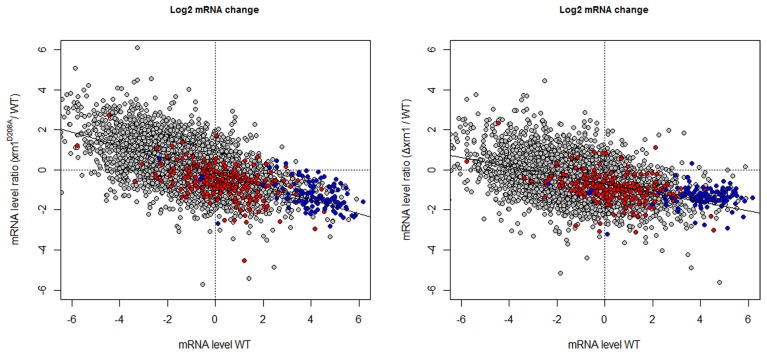
**Changes in the mRNA level in *xrn1* mutants inversely correlate with the mRNA level.** Plots of the ratios between the mutant and the wt mRNA level against the mRNA level in the wt. See Figure 1 for other details.

The predictable effect of the HL increase would be a parallel increase in the mRNA amount (concentration) in the *xrn1* mutants. However, not only did the levels of most mRNAs not increase, but also the levels of many of them slightly decreased, especially in the deletion mutant (Figure 3 and Haimovich et al., 2013). Moreover, a negative increased tendency in (mRNA) was noted with regard to the mRNA steady-state mRNA levels in the wt (wild type; Figure 3).

### Xrn1 deletion reduces transcription rates genome-wide, but with a strong bias to translation-related genes

**Figure 4:**
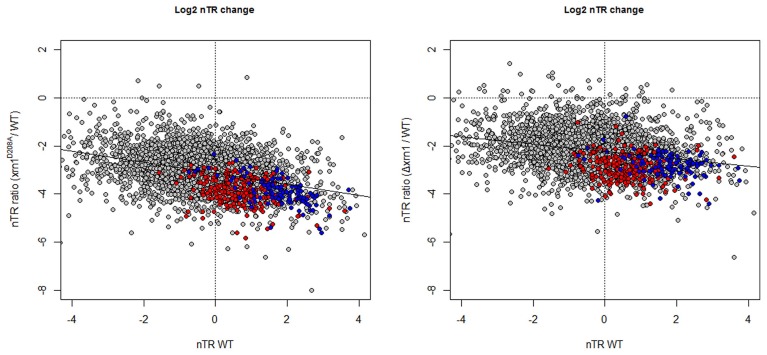
**Changes in the nTR level in *xrn1* mutants inversely correlate with nTR.** Plots of the ratios between mutant and wt nTR against nTR in the wt. nTR was determined as in García-Martínez et al. (2004). The median nTR was defined arbitrarily as 1 (0 on the Log_2_ scale). Note that the ratios obtained for most genes are negative, indicating that the transcription in the mutant was lower than in the wt counterpart. See Figure 1 for other details.

The analysis of the nTR by GRO shows that most genes had a lower nTR level in the cells carrying the disrupted *xrn1*. The drop in the nTR was greater for not only the genes with a higher nTR in wt (Figure 4), but also for the point mutant, as we previously noted (Haimovich et al., 2013).

The analysis of the overrepresented GOs among those genes with a more marked nTR decrease showed a strong bias toward the processes affecting the different protein synthesis steps: rRNA synthesis, modification and transport and translation-related (Table S1; Figure 2B). All these GO categories are closely linked to the genes belonging to the RiBi (Ribosome Biogenesis) regulon (Jorgensen et al., 2004) or to ribosomal protein (RP) genes. Similar results (not shown) were obtained when using data from Sun et al. (2013) who determined mRNA synthesis rates using a different method, arguing that there is no bias inherent to nTR determination by the GRO method. Thus, these GO categories are related to, but are not all identical to, those found in the HL change.

As expected from the previously discussed mRNA HL results, a slight, but statistically significant inverse correlation, was observed between the drop in the nTR and the increase in the HL (Figure S1), as we previously noted in a previous paper (Haimovich et al., 2013). Hence, the synthesis of unstable mRNA is affected more by Xrn1 disruption or deletion than stable mRNAs. The effect on RP and RiBi genes is, however, stronger than the expected for their nTR because both groups are clearly below the tendency line (see Figure 4).

The responsiveness of the yeast genes in the nTR or the HL when Xrn1 activity is lacking seems to be correlated. There is a strong overlap between genes most affected in TR and in HL (Figure 2E). Therefore in a single study, we analyzed combined nTR+HL responsiveness. Genes were ordered and divided into quintiles for the lowered nTR and in the increased HL (thiolutin experiment) due to Xrn1 disruption. The most and least marked effect on either was qualified as 5 and 1, respectively. Those genes with 8 points or more in the sum of both were, therefore, strongly affected in both transcription and mRNA degradation, whereas those with 3 points or less were classified as less affected. The results are summarized in Table S2. It should be noted that we analyzed only 1915 genes because the HL was taken from the thiolutin experiment, which provides fewer data, and not from the indirect calculation in order to avoid mathematical linkage, as previously explained. In spite of the low number of genes, it is very clear that the strongly affected genes belong primarily to the GO categories involved directly in translation or the indirectly related ones, such as RiBi or ribonucleotide biosynthesis (mainly required for rRNA biogenesis) (Table S1; Figure 2C).

It is also noteworthy that the GO categories (and genes) with the best nTR or DR responsiveness to Xrn1 inactivation are similar to those with a high nTR and a low HL (higher degradation rate, DR) in the wt cells during exponential growth (García-Martínez et al., 2007). That is to say, those mRNAs in the wt with a high turnover (synthesis + degradation) rate, because their nTR and/or DR is/are high, are those most affected by Xrn1 inactivation (Figure S2).

### Xrn1 acts at both the initiation and elongation levels in transcription

The effect of Xrn1 inactivation on nTR led us to study the mechanism that this moonlighting protein uses to activate transcription. In a previous publication, we showed that Xrn1 binds the gene promoters and 5' flanks of the transcribed regions, and that it differently affects genes with regard to their length (Haimovich et al., 2013). This work extends those studies by searching for biases in the type of genes bound by Xrn1.

**Figure 5:**
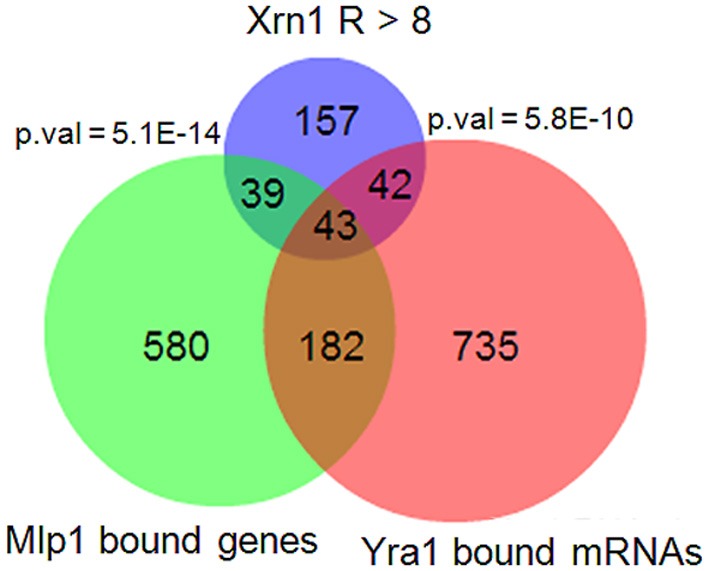
**Overlapping between Xrn1-synthegradon genes with genes bound by Mlp1 and the mRNAs bound by Yra1.** The list of Xrn1-synthegradon genes (responsiveness index >8, this work) was crossed with the genes bound by Mlp1 (Casolari et al., 2004) and with mRNAs bound by Yra1 (Hieronymus and Silver, 2003). The overlaps are statistically significant (*p*-values for hypergeometric tests are shown). The overlaps with other Cse1 nuclear pore proteins studied in the same paper were also significant (Figure S4), but not with Nup60.

A preliminary analysis revealed no clear difference in the nTR, HL or RA effect between the TATA and the TATA-like genes (Figure S3). Previously, we determined Xrn1 binding to chromatin by the ChIP-exo technique to find that Xrn1 bound many genes. Among these genes, 535 exhibited efficient binding (above an arbitrary threshold), and preferably, but not exclusively, at the promoters. The analysis of these 535 genes showed that they were also enriched in the translation-related categories (Table S1, Figure 2D). A statistically significant overlap was observed (Figure S4A) among the genes bound by Xrn1, and the genes bound by the NPC-related proteins Mlp1 and Cse1 (Casolari et al., 2004), and those whose mRNAs were bound by Yra1 (Hieronymus and Silver, 2003, not shown). The statistical significance was even higher when we compared genes having a high (>8) responsiveness index (Figure 5; Figure S4B). We also examined the sequences of the Xrn1-bound promoter and found enrichment in sequence motif AAAAARAAAAA. A similar motif was found by Casolari et al. (2004) in the genes interacting with Mlp1, Mlp2, and Nic96.

Therefore, it seems that translation-related genes are bound by Xrn1p, whose transcription especially decreases, and their cytoplasmic mRNAs were stabilized especially by Xrn1 inactivation. We can conclude that Xrn1p affects the synthesis and degradation of most mRNAs, but it is especially important as a synthegradase for those mRNAs whose products are required for cell growth and proliferation, such as RiBi, ribosomal proteins, translation factors and metabolic factors.

**Figure 6:**
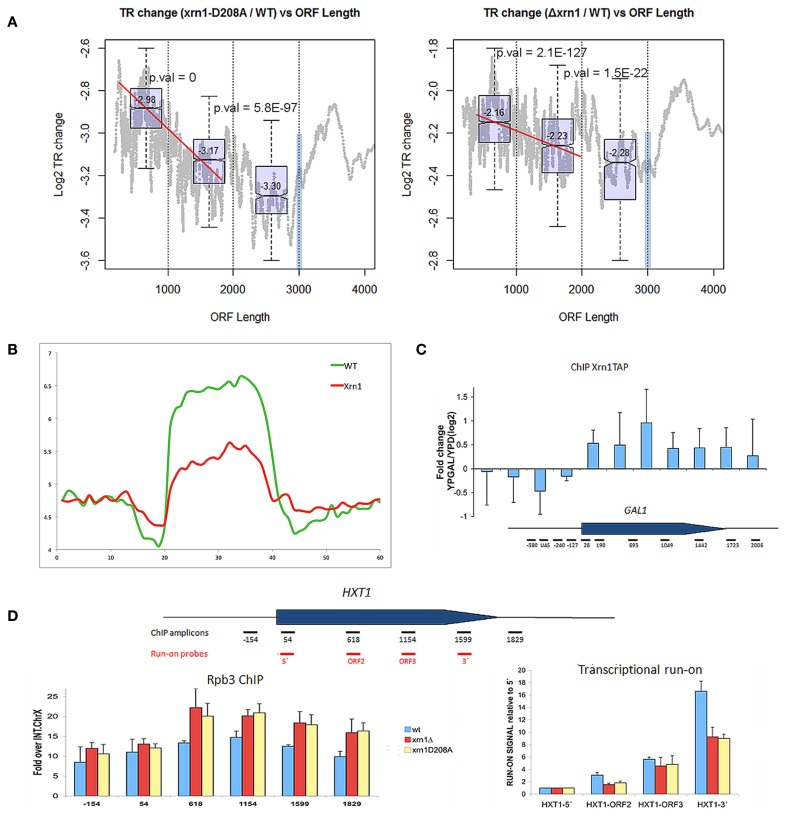
**nTR decreases in *xrn1* mutants. (A)** Changes in nTR of the *xrn1* mutants are plotted against ORF length. A decreasing tendency is clearly seen for the genes between 300 and 2000 bp. This is result is reinforced with the box-plots (distribution and median values and *p*-values for the difference between two consecutive groups) for the genes situated in each of the three groups of the distribution (0-1 kb, 1-2 kb, 2-3 kb). At 3 kb, a blue bar shows the artifact caused by the displacement toward 3′ during the run-on, which provokes a bias against long genes (see Pelechano et al., 2010). However, the ratio between *xrn1* and wt should not be affected. This challenges the interpretation of the graphs, although it indicates that the behavior of the final 1 kb differs from the rest. **(B)** Presence of active RNA pol II along a “metagene” at a high resolution using BioGRO average for the whole yeast genome. On the Y-axis, the arbitrary fluorescence value indicates elongating RNA pol II density. On the X-axis region, from 0 to 20 represents 1 kb upstream of the gene on a real scale, and the region from 20 to 40 represents the gene region from TSS to TTS on a relative scale (all gene lengths become normalized), while the region from 40 to 60 represents 1 kb after TTS on a real scale. The relative height of the plateau in the wt and Δ*xrn1* mutant depicts the nTR along the whole average gene. **(C)** Binding Xrn1-TAP to the *GAL1* gene, as measured by ChIP in the indicated amplicons. The ratio between the ChIP signal in YPGal, where *GAL1* is transcriptionally active, and YPD, where *GAL1* is repressed, is shown. **(D)** Distribution of RNA pol II across the *HXT1* gene in the YPD-grown wild-type cells and in the isogenic *xrn1* mutants. Total RNA pol II occupancy was followed by anti-Rpb3 ChIP. Active RNA pol II was followed by transcriptional run-on. Error bars indicate standard deviation.

Finally, we investigated the effect of Xrn1 deletion on transcription elongation. In our previous paper, we described that the transcription of long genes is affected by Xrn1 disruption more than shorter ones. Here we show that a negative slope in the nTR is evident especially for genes shorter than 1.5–2 kb (Figure 6A). We also performed the run-on experiment following a newly developed non- radioactive variant that uses Biotin-UTP as a precursor for run-on and Affymetrix tiling arrays for high resolution mapping (Jordán-Pla et al., in preparation). This method, called BioGRO, also permits the study of the intragenic distribution of elongating RNA polymerases. Using BioGRO, we found that the average BioGRO map along genes bodies shows that the level of active RNA pol II, from the start site to the end, is lower in the Δ*xrn1* than in the wt (Figure 6B), which is compatible with the reduction in transcription initiation postulated using ChIP-exo data in Xr1p binding (Haimovich et al., 2013). Moreover, we also found a bias for longer genes, and even for genes shorter than 1.5 kb (not shown). To verify this effect on elongation using individual genes, we analyzed two single yeast genes: *GAL1*, a gene that is repressed strongly in glucose and is activated in galactose; *HXT1*, a more active gene in glucose. Figure 6C shows that the Xrn1 protein is cross-linkable to the transcribed region of *GAL1* in a transcription-dependent manner. This result is consistent with the elongation defect that we reported for *GAL1* transcription in galactose-containing medium (Haimovich et al., 2013). Since all the other experiments of this work have been carried out in glucose-containing medium, we analyzed elongation through *HXT1*, a gene that reaches the maximum expression level under glucose conditions. We found that the distribution of active RNA pol II, detected by run-on, is strongly affected in *xrn1* mutants, and that it displays a progressively decreasing run-on across the gene body (Figure 6D). This decreased run-on was parallel to the increased RNA pol II occupancy, as measured by ChIP (Figure 6D). This combination of a lower run-on signal and greater RNA pol II occupancy is indicative of higher levels of RNA pol II backtracking during elongation (Rodríguez-Gil et al., 2010; Gómez-Herreros et al., 2012). In line with this, it seems significant that the gene categories which are more responsive to the *XRN1* mutation, like those related to RP genes, constitute regulons that regulate their degree of backtracking in response to physiological changes (Pelechano et al., 2009). All these results support a role for Xrn1 in transcription elongation.

## Discussion

In a previous paper (Haimovich et al., 2013), we found that, in addition to its known function in the degradation of cytoplasmic mRNAs, Xrn1 (as well as other mRNA decay factors) functions as a transcription factor. We found that Xrn1 physically associates with many genes, and that it prefers to bind promoters of highly transcribed genes. Moreover, its inactivation provokes a substantial reduction in the nTRs that counteract the decrease in DRs. We called Xrn1 synthegradase because of its dual activity in synthesis and degradation. Consistently with its role as a synthegradase, we found that Xrn1 shuttles between the nucleus and the cytoplasm. The nuclear location of plant Xrn1 has previously been reported; it is proposed to be related to its role in snoRNA or tRNA processing (Nagarajan et al., 2013). Therefore, this moonlighting protein may perform several different nuclear and cytoplasmic roles.

In this paper, we have further explored our previous results and carried out new experiments to confirm the role of Xrn1 in transcription. Our results demonstrate that the specificity of Xrn1 is broad as it seems to act on most genes and mRNAs. However, transcription of the most actively transcribed genes is more sensitive to the inactivation of Xrn1 than other genes. Interestingly, the genes targeted by Xrn1 tend to encode unstable mRNAs. These two features suggest a linkage between the role of Xrn1 in transcription and mRNA decay. These results probably reflect the more pressing need for tight mRNA homeostasis for many abundant mRNAs (which tend to have higher TRs and/or higher DRs). The structural components of the ribosome (RP) and the translation process itself, which are among the most highly transcribed genes (Pelechano et al., 2010), are preferentially bound by Xrn1. Indeed, transcription of these genes is highly dependent on Xrn1. However, genes whose transcription is of medium level are also preferred clients of Xrn1. The most notable are those encoding ribosome biogenesis factors (RiBi), whose transcription is highly dependent on Xrn1 and their mRNAs are very strongly stabilized in *xrn1* mutants. The combined effects of both (as seen in the “responsiveness” index or in the [mRNA] change, Table S1) produce a general effect on the translation-related GO categories (i.e., RP + RiBi). The Xrn1 activities on transcription and degradation rates are not necessarily of the same intensity, what is reflected in the (negative) slope in mRNA change vs. [mRNA](Figure 3), and in specific deviations from the cloud tendency line: RiBi genes are slightly below and RP genes are slightly above it (Figure 3, red and blue dots).

Cytoplasmic yeast mRNAs are degraded by two major pathways (Parker, 2012). We found that there is an inverse relationship between mRNA HL and the effect that Xrn1 has on this HL (Figure 1). It is possible that the very unstable mRNAs are degraded preferentially by Xrn1, the moderately unstable are degraded by both Xrn1 and the exosome, and stable mRNAs (that are the minority) by the exosome.

The results provided in this paper confirm those previously obtained (Haimovich et al., 2013) on the dual role of Xrn1 in transcription initiation and elongation. They provide three additional important results. First, the effect of Xrn1 disruption on transcription seems to be more critical for highly transcribed genes (Figure 4). This is not a trivial consequence of the higher nTR because the dependence on nTR in *xrn1* mutants is with the ratio of decrease not just with the difference with wt. Consistent with a role of Xrn1 in PIC formation is the observation that Xrn1 prefer to bind ~30 bp upstream of transcription start sites (Haimovich et al., 2013), where PIC is assembled. The dual role of Xrn1 in mRNA synthesis and decay classifies it as a synthegradases (Bregman et al., 2011), whose selectivity, demonstrated here, might help coordinating the expression of the protein synthetic machinery. We propose to name the most affected genes “Xrn1 synthegradon” because they are regulated by Xrn1 both as pre- and post-transcriptional regulons. Interestingly, Xrn1 is also involved in the maturation of the rRNAs (see Nagarajan et al., 2013 for review). Thus, its critical role in the expression of Xrn1 synthegradon, along with its role in rRNA maturation places Xrn1 as a critical factor of protein biosynthetic machinery. It would be interesting to examine a possible linkage between the roles of Xrn1 in rRNA maturation and its roles in the expression of the Xrn1 synthegradon.

Second, the reduction in elongating RNA pol II is observed along the affected genes from the very beginning (Figure 6B). Moreover, the binding of Xrn1 is specific to transcribed chromatin regions, as can be seen in its preferential binding with active vs. inactive *GAL1* (Figure 6C). Importantly, Xrn1 seems to keep the elongating complex in a transcription-competent state, because if Xrn1 is lacking a larger portion of chromatin-bond pol II molecules is inactive, as evident by the [total RNA pol II/active RNA pol II] ratio in both *GAL1* (Haimovich et al., 2013) and *HXT1* (Figure 6D). These results suggest that Xrn1 helps preventing pol II backtracking during elongation.

Third, Xrn1-syntegradon genes seem to encode transcripts that are exported by Yra1, Mlp1, and Cse1 (Figure 5; Figure S4). It is worth noting that Yra1, which is mainly a nuclear factor, has been reported to physically interact with mRNA decay factors Pat1 and Xrn1 in two different screenings (Kashyap et al., 2005; Krogan et al., 2006, respectively). Our results and these interactions may represent a linkage between the decaysome and Yra1 in the nucleus and reinforce the synthegradon concept we propose here.

In *Arabidopsis*, the absence of XRN4 (the presumed functional counterpart of yeast *XRN1*) leads to alterations in the levels of a number of mRNAs, mainly encoding nucleic acid binding proteins with a role in transcription, RNA stability and translation (Rymarquis et al., 2011). Remarkably, these GOs are subpopulation of the Xrn1 synthegradon (Table S1). The fact that these mRNAs are increased in the mutant *Arabidopsis* but not in the mutant yeast suggests that the relative contribution of Xrn1 on their synthetic and decay is different in the two organisms. It is worth noting that some transcripts decrease in response to Xrn1/XRN4 deletion in both yeast (Kim and Kim, 2002; our study) and *Arabidopsis* (Rymarquis et al., 2011). Thus if XRN4 is also a synthegradase, like the yeast Xrn1, it might be regulated differently. We note that the average mRNA HL of unicellular organisms, like yeasts, is substantially shorter than that of multicellular organisms (Pérez-Ortín et al., 2013). No wonder, then, that Xrn1 and XRN4 might be regulated differently.

In light of the global roles of Xrn1 in nTR and DRs, it is surprising that this protein is not essential in *S. cerevisiae*, *Drosophila* and *Arabidopsis.* However, this can be reconciled if we assume that all the pathways in which Xrn1 participates are highly parallel. Its roles in transcription stimulation and mRNA decay have alternative pathways that can substitute it. Therefore, its novel role as a synthegradase might be critical for fitness of the organism under ever-changing environment, but is not essential under optimal conditions (Geisel, 2011; Fierst and Phillips, 2012; Rest et al., 2013).

## Author contributions

Daniel A. Medina and José E. Pérez-Ortín designed, performed and analyzed the GRO and thiolutin shut-off experiments and all the bioinformatics analyses. Antonio Jordán-Pla performed BioGRO experiments. Mordechai Choder suggested some tests and comparisons. Gonzalo Millán-Zambrano and Sebastián Chávez designed, performed and analyzed the Pol II and Xrn1 ChIP and run-on experiments of *GAL1* and *HXT1* in glucose and galactose. José E. Pérez-Ortín wrote the manuscript. Mordechai Choder and Sebastián Chávez critically read and edited the manuscript.

### Conflict of interest statement

The authors declare that the research was conducted in the absence of any commercial or financial relationships that could be construed as a potential conflict of interest.

## Supplementary material

Figure S1 **Changes in nTR in the *xrn1* mutants inversely correlate with their mRNA stability changes**. Plots of the ratios between the mRNA levels in the mutant cells and those in the wt against the ratios between mRNA HL in the mutant cells and those in the wt. The cloud shows 1915 data for the genes with confident HL measures in a shut-off with thiolutin in both the wt and mutants. See Figure 1 for further details and for the color code.

Figure S2 **Genes that are more affected in nTR or HL by the Xrn1 mutation strongly correlate with those with the highest TR/HL ratio**. The genes exhibiting a sharp HL decrease in *xrn1*-D208A or Δ*xrn1* significantly overlap those 2070 genes with the highest nTR/HL ratio (2070), according to García-Martínez et al. (2007). The *P*-values of the hypergeometric tests are shown.

Figure S3 **TATA box-containing genes have similar nTR, mRNA levels and mRNA stability changes in the *xrn1* mutants as TATA-like genes**. Box plots for the distribution and median values of the TATA or TATA-like genes for nTR determined by GRO **(A)**, mRNA levels determined by macroarray hybridization **(B)** and mRNA HL determined by transcription shut-off with thiolutin **(C)**. The *t*-test results are shown above each comparison. Although some of the *t*-tests provide significant *p*-values the comparisons show that the changes in both the TATA-containing and TATA-like genes at the mRNA, nTR or HL levels in the *xrn1* mutants are not biologically relevant.

Figure S4 **Overlapping between the Xrn1-dependent genes and genes bound by Cse1**. The list of genes bound by Cse1 and Mlp1 (Casolari et al., 2004) was crossed with Xrn1-bound **(A)** or Xrn1-synthegradon **(B)** genes. The overlaps are statistically significant (the *P*-values for the hypergeometric tests are shown). See Figure 5 for further details.

Table S1 **Statistically enriched GO categories in the *xrn1* mutants for nTR, HL, and (mRNA) and for the Xrn1-bound genes. Red categories are those not related to protein biosynthesis**.

Table S2 **List of genes classified according to their nTR and HL changes in the *xrn1* mutants**. The responsiveness index is also provided.

## Abbreviations

ChIP: chromatin immunoprecipitation
DR: degradation (decay) rate
HL: mRNA half-life
[mRNA]: cytoplasmatic mRNA concentration
nTR: nascent transcription rate
RNA pol: RNA polymerase
GRO: Genomic Run-On
BioGRO: Biotin-GRO
GO: Gene Ontology
NPC: nuclear pore complex
wt: wild type
PIC: pre-initiation transcription complex
RP: ribosomal proteins
RiBi: Ribosome Biogenesis
