# Supplementary figures and images for: Cytoplasmic 5′-3′ exonuclease Xrn1p is also a genome-wide transcription factor in yeast
Source: Front Genet. 2014 Feb 6;5:1. doi: 10.3389/fgene.2014.00001 (PMC3915102; doi:10.3389/fgene.2014.00001)

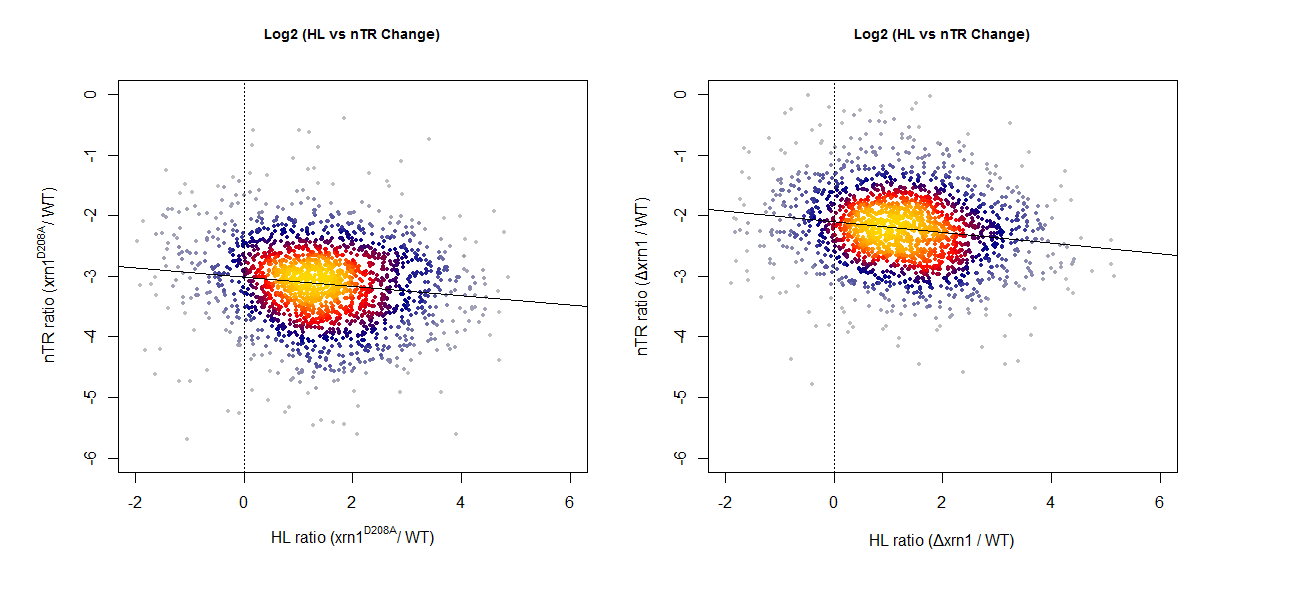

Supplement: Figure S1 — Changes in nTR in the xrn1 mutants inversely correlate with their mRNA stability changes. Plots of the ratios between the mRNA levels in the mutant cells and those in the wt against the ratios between mRNA HL in the mutant cells and those in the wt. The cloud shows 1915 data for the genes with confident HL measures in a shut-off with thiolutin in both the wt and mutants. See Figure 1 for further details and for the color code. [file Presentation1.ZIP › 74347_Pérez-Ortín_Suppl_Figure_1.TIF]

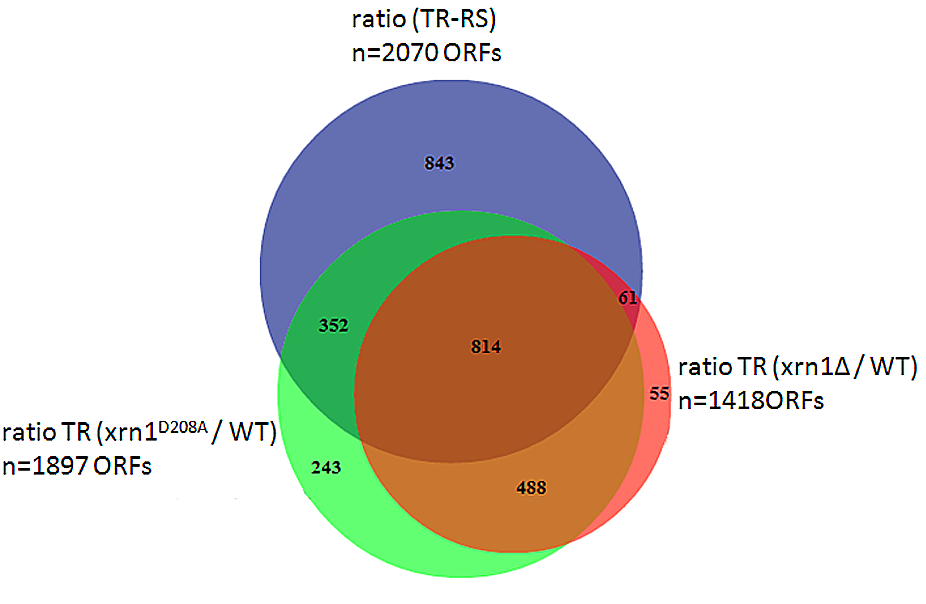

Supplement: Figure S1 — Changes in nTR in the xrn1 mutants inversely correlate with their mRNA stability changes. Plots of the ratios between the mRNA levels in the mutant cells and those in the wt against the ratios between mRNA HL in the mutant cells and those in the wt. The cloud shows 1915 data for the genes with confident HL measures in a shut-off with thiolutin in both the wt and mutants. See Figure 1 for further details and for the color code. [file Presentation1.ZIP › 74347_Pérez-Ortín_Suppl_Figure_2.TIF]

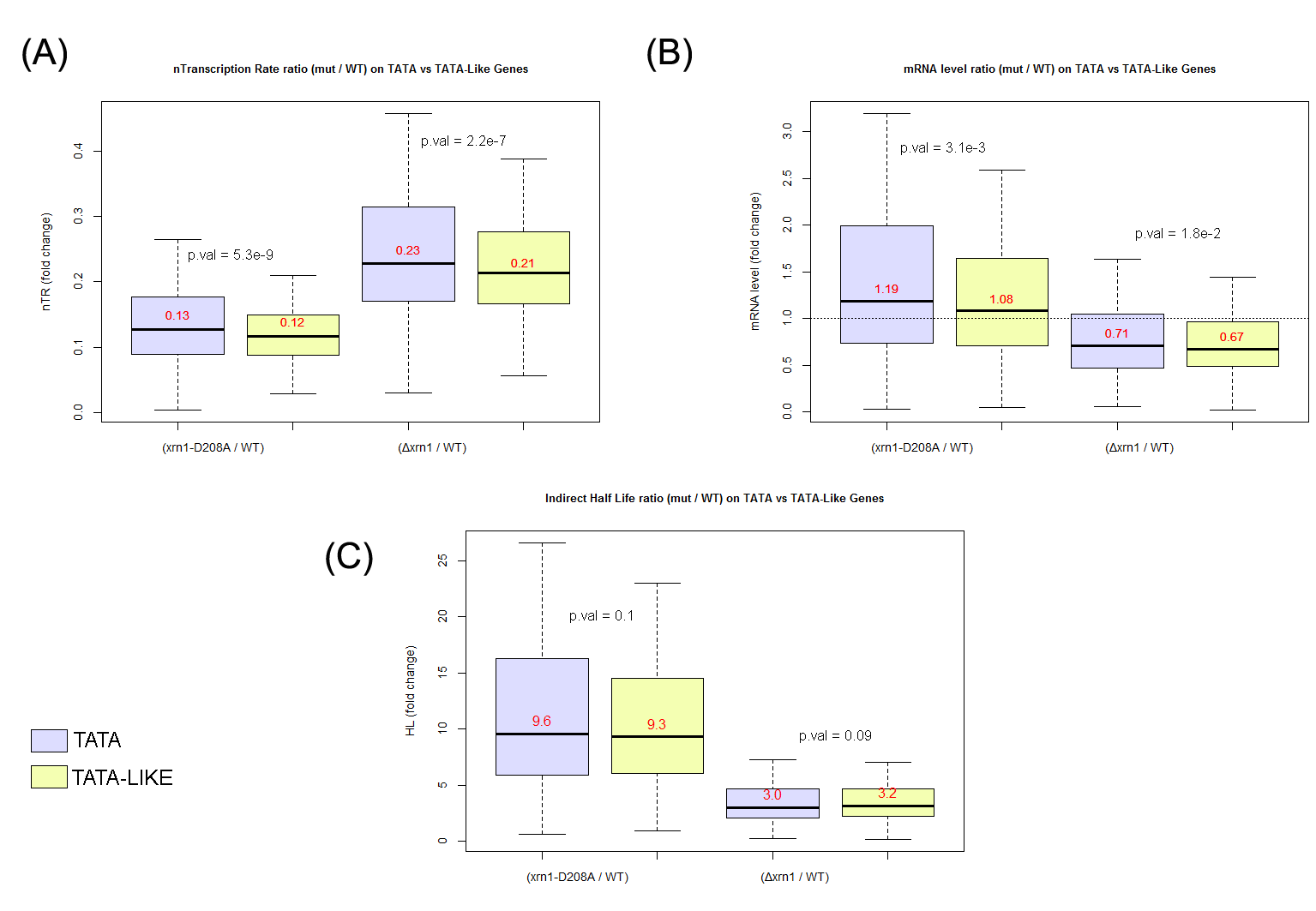

Supplement: Figure S1 — Changes in nTR in the xrn1 mutants inversely correlate with their mRNA stability changes. Plots of the ratios between the mRNA levels in the mutant cells and those in the wt against the ratios between mRNA HL in the mutant cells and those in the wt. The cloud shows 1915 data for the genes with confident HL measures in a shut-off with thiolutin in both the wt and mutants. See Figure 1 for further details and for the color code. [file Presentation1.ZIP › 74347_Pérez-Ortín_Suppl_Figure_3.TIF]

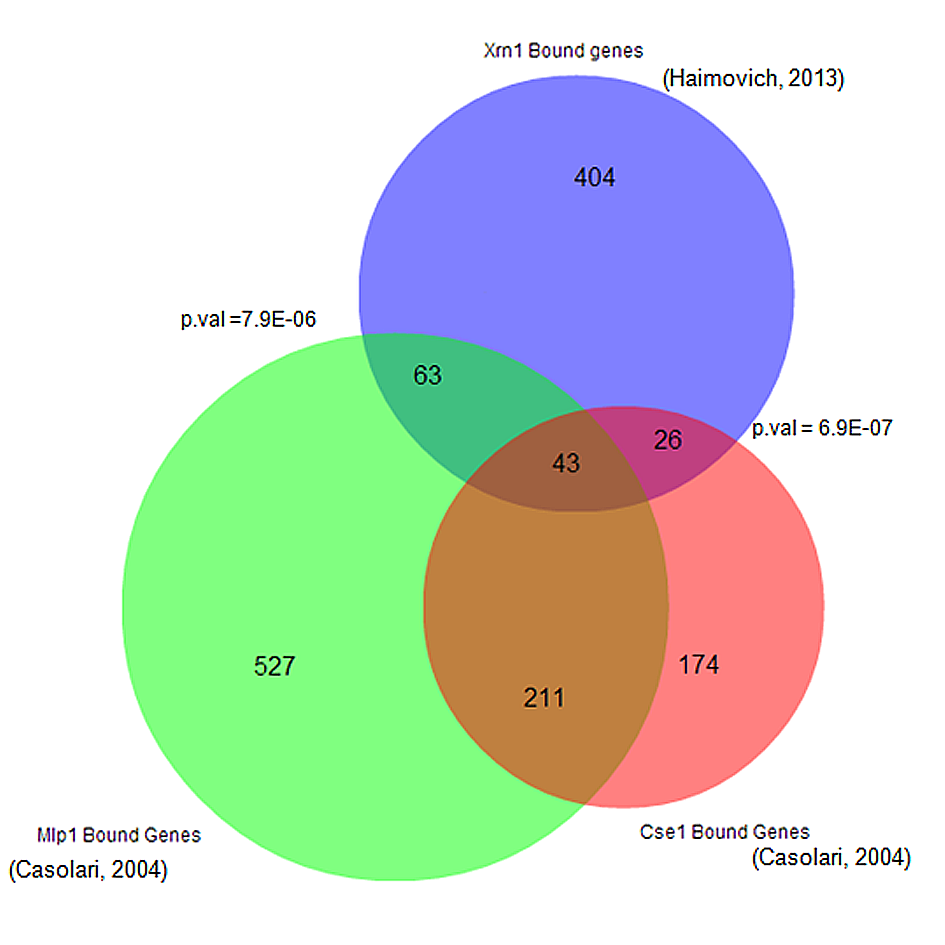

Supplement: Figure S1 — Changes in nTR in the xrn1 mutants inversely correlate with their mRNA stability changes. Plots of the ratios between the mRNA levels in the mutant cells and those in the wt against the ratios between mRNA HL in the mutant cells and those in the wt. The cloud shows 1915 data for the genes with confident HL measures in a shut-off with thiolutin in both the wt and mutants. See Figure 1 for further details and for the color code. [file Presentation1.ZIP › 74347_Pérez-Ortín_Suppl_Figure_4.TIF]
